# EGFR/Vimentin/Folic Acid磁球检测肺癌循环肿瘤细胞初探

**DOI:** 10.3779/j.issn.1009-3419.2020.103.05

**Published:** 2020-05-20

**Authors:** 国雷 李, 赟 王, 国梁 谭, 远 刘, 曌 徐, 浩 冯, 伟 兴, 志峰 徐

**Affiliations:** 1 050051 石家庄, 河北省中医院外一科 The First Surgery Department, Hebei Provincial Hospital of Chinese Medicine, Shijiazhuang 050051, China; 2 050051 石家庄, 河北医科大学第三医院超声医学科 Department of Medical Ultrasonics, The Third Hospital of Hebei Medical University, Shijiazhuang 050051, China

**Keywords:** 肺肿瘤, 循环肿瘤细胞, EGFR, Vimentin, 叶酸, 免疫磁球, Lung neoplasms, Circulating tumor cells, EGFR, Vimentin, Folic acid, Immune magnetic sphere

## Abstract

**背景与目的:**

循环肿瘤细胞(circulating tumor cell, CTC)在肺癌的筛查及预后方面发挥着重要的作用, 但较低的CTC分离效率和特异性对其临床应用有着明显的制约, 本研究旨在探讨非小细胞肺癌(non-small cell lung cancer, NSCLC)患者CTC的新型高效分离方法, 以期达到对NSCLC的早期诊断的目的。

**方法:**

采用薄膜法制备表皮生长因子受体(epidermal growth factor receptor, EGFR)、波形蛋白(Vimentin)和叶酸(folic acid, FA)三种免疫脂质磁球, 表征后通过细胞系进行分选方案的探索, 构建对NSCLC CTC的最优分选方案, 初步研究了其在临床上的应用价值。

**结果:**

EGFR、Vimentin和FA磁球磁球单独和联合使用对肺癌细胞株的平均捕获效率分别为78.0%、79.0%、82.0%和91.0%;在60例肺癌患者中, 以每7.5 mL血液2个CTC为cutoff值, EGFR、Vimentin、FA磁球单独和联合使用阳性率分别为65.0%、33.3%、93.3%和100.0%, 同时发现联合使用三种磁球检出的CTC数量与临床分期具有相关性(*P* < 0.05)。

**结论:**

联合使用三种磁球可以分离EGFR+、Vimentin+和FA+表达且形态完整的CTC, 有利于的CTC相关下游分析, 本研究提供了一种提高NSCLC CTC捕获效率的新方法, 且验证了捕获的CTC计数方法可用于肺癌的辅助诊断。

## Abstract (en)

**Background and Objective:**

Circulating tumor cells (CTC) play an important role in the screening and prognosis of lung cancer, but the low efficiency and specificity of CTC isolation obviously restrict its clinical application. The purpose of this study is to explore a new and efficient isolation method of CTC in patients with non-small cell lung cancer (NSCLC) in order to achieve the purpose of early diagnosis of NSCLC.

**Methods:**

Three kinds of immunolipid magnetic spheres of epidermal growth factor receptor (EGFR), vimentin and folic acid (FA) were prepared by thin film method. After characterization, the sorting scheme of cell line was explored, the optimal sorting scheme of NSCLC CTC was constructed, and its clinical application value was studied.

**Results:**

The average capture efficiency of EGFR, Vimentin and FA magnetic spheres used alone and in combination to lung cancer cell lines was 78%, 79%, 82% and 91%, respectively. In 60 patients with lung cancer, using 2 CTC per 7.5 mL blood as cutoff value, the positive rates of EGFR, Vimentin and FA magnets used alone and in combination were 65.0%, 33.3%, 93.3% and 100%, respectively. It was also found that the number of CTC detected by combined use of the three magnetic spheres was correlated with clinical stages (*P* < 0.05).

**Conclusion:**

The combination of three kinds of magnetic spheres can separate EGFR+, Vimentin+, FA+ expressed CTC, which is beneficial to the downstream analysis of CTC correlation. This study provides a new method to improve the efficiency of NSCLC CTC capture, and verifies that the captured CTC counting method can be used in the auxiliary diagnosis of lung cancer.

癌症的晚期经常发生转移, 肿瘤的转移导致约90%的癌症死亡, 使其成为了癌症治疗的焦点^[1]^。肿瘤细胞可以从原发部位迁移到循环系统(例如：血液、淋巴液和脑脊液等)然后扩散到其他器官。此时癌细胞亚群离开原发肿瘤, 通过血液等循环迁移并定植到新的脏器组织形成新的肿瘤块^[2, 3]^。因此, 早期识别通过血液循环传播的癌细胞, 即循环肿瘤细胞(circulating tumor cell, CTC), 及时准确地检测稀有和极少数CTC, 对于癌症治疗的成功和改善患者的生存至关重要。CTC的存在与预后不良密切相关^[4-6]^, 然而, 血液中的CTC的丰度极低, 在转移的早期阶段检测CTC是一项挑战, 从患者获得的7.5 mL血液样本中识别和捕获极少数的肿瘤细胞存在一定的困难^[2, 7]^。

以往常用于检测癌症患者血液中CTC的技术较成熟的为CellSearch^[8]^, 其他各种捕获技术, 包括免疫磁珠、功能化微纳米结构、活体流式细胞仪等也都正在发展与应用中^[9-13]^。其中, 基于免疫化学的磁性纳米颗粒可以高效率和高选择性地识别和捕获全血中的CTC。CellSearch是基于抗体包被的磁性纳米颗粒连接上皮细胞黏附分子(epithelial cell adhesion molecule, EpCAM)捕获CTC的技术。考虑到基于抗体的CTC捕获技术的局限性, 例如不能够捕获缺乏EpCAM蛋白的CTC或已经发生上皮间质转化的CTC^[14]^, 因此, 捕获此类CTC需要其他蛋白抗体修饰的磁珠才能够实现。

叶酸(folic acid, FA)已经成为非小细胞肺癌(non-small cell lung cancer, NSCLC)患者的一个重要的潜在药物靶点^[15, 16]^。有研究^[17]^发现在NSCLC患者中, FR表达上调约75.7%。表皮生长因子受体(epidermal growth factor receptor, EGFR)蛋白在NSCLC中的阳性表达率为53%, 在性别及有无淋巴结转移之间阳性表达率有显著差异, 而在年龄、病理类型、肿瘤分化程度、吸烟史及临床分期之间无显著差异^[18]^。肿瘤细胞中的波形蛋白(Vimentin)可呈现过量表达, 在NSCLC中, 高表达的Vimentin蛋白可作为较差预后的独立观测指标; 低表达的Vimentin则是患者良好生存质量的独立预测因子^[19]^。在这里, 我们分别制备了EGFR、Vimentin、FA三种免疫脂质体的磁性纳米颗粒系统, 用于NSCLC细胞的特异性靶向快速分离检测, 比较了分别使用EGFR、Vimentin、FA三种磁球对NSCLC CTC的捕获效率及同时使用三种磁球的捕获效率, 并在真实世界中利用它们对临床NSCLC患者血样进行CTC捕获, 初步研究了其在临床上的应用价值。

## 1 材料与方法

### 1.1 标本来源

收集本院2017年9月-2019年9月NSCLC患者血标本60例, 样本采集方法是用医用抗凝采血管采集患者外周血7.5 mL, 抗凝剂为EDTA·K2。患者年龄36岁-75岁, 中位年龄53.5岁, 平均年龄55岁。同时招募20名健康志愿者, 收集其全血作为实验的对照。所有选取的病例均履行告知义务并签署知情同意书。

### 1.2 细胞株

A549细胞株、HCC827细胞株、NCI-H1650细胞株、NCI-H3122细胞株均购自ATCC细胞库。细胞中用含10%新生牛血清的RPMI-1640培养液, 37 ℃, 5%CO_2_, 恒温培养箱内培养。

### 1.3 材料与仪器

DMEM培养液、胎牛血清、胰蛋白酶购于Gibco公司。CD45-PE购自eBioscience公司。CK-FITC和脂质磁球购自举康(上海)生物科技有限公司。DAPI染色液购自碧云天生物技术有限公司。EGFR抗体、Vimentin、FA购自猎源(上海)生物医药科技有限公司。普鲁士蓝染色试剂盒购自Solarbio公司。二硬脂酰基磷脂酰乙醇胺-聚乙二醇(DSAPC-APCG)购自上海晟纳实业有限公司。胆固醇(Chol)、二氯甲烷、及其他常用试剂均购自国药公司。BI-90Plus激光粒度仪/Zeta电位仪购自美国布鲁克-海文公司。XL-30型环境扫描电子显微镜购自荷兰PHILIPS公司。LDJ9600-1型VSM磁性能测试仪购自美国数字仪器公司。OLYMPUS B×61型荧光显微镜购自日本奥林巴斯公司。超声波细胞粉碎机型号JY92-IIDN, 旋转蒸发仪型号XD-52AA均购自上海般诺生物科技有限公司。

### 1.4 免疫脂质磁球的制备

采用薄膜法制备EGFR免疫脂质磁球, 具体制备过程及试剂用量参阅参考文献^[20, 21]^。将PEG-DSPE、胆固醇、DOPC、GHDC、HQCMC、Fe_3_O_4_溶液共溶于二氯甲烷中, 同时加入浓度为0.1 mol/L的PBS, pH为7.4, 使用探针式超声波仪对混合溶液进行超声振荡, 功率为27%, 超声2 s, 间隔1 s, 总时间6 min, 温度25 ℃, 使其完全乳化, 得到脂质磁球(LMB)溶液。取0.6 mg EGFR多肽溶于10 mL异丙醇中, 分别加入偶联剂1-ethyl-3-(3-dimethylaminopropyl)carbodiimide(EDC)和N-hydroxysuccinimide(NHS), 保持4 ℃匀速搅拌24 h, 即可获得EGFR修饰的脂质磁球。Vimentin和FA脂质磁球的制备同EGFR脂质磁球的制备。

### 1.5 免疫脂质磁球的表征

免疫脂质磁球的粒径电位的测定采用BI-90Plus激光粒度仪/Zeta电位仪检测, 取10 μL样品在1 mL蒸馏水中稀释后, 用于粒径电位测试。免疫LMB的形态通过原子力显微镜(atomic force microscope, AFM)观察, 取10 μL样品在1 mL蒸馏水中稀释后, 取50 μL涂于载玻片上, 待干后进行测量。免疫LMB紫外吸收光谱检测通过紫外分光光度计检测, 取10 μL样品在1 mL蒸馏水中稀释后, 直接进行测量。磁球捕获A549细胞的普鲁士蓝染色通过普鲁士蓝染色试剂盒, 按其说明书操作进行。

### 1.6 细胞培养

本研究应用的肺癌细胞系A549细胞、HCC827细胞、NCI-H82细胞、NCI-H146细胞在DMEM完全培养液中常规培养, 完全培养液中含有10%的胎牛血清(Fetal bovine serum, FBS)。培养条件为湿润状态下37 ℃和5%CO_2_。培养液用量：35 mm培养皿为2 mL培养液, 60 mm培养皿为3 mL培养液, 10 cm培养皿为8 mL培养液。细胞的冻存与复苏：将细胞用适量的1.25%的胰酶消化, 待细胞变圆、尚未漂起时候加入适量完全培养液并反复吹打形成细胞悬液。按照细胞悬液:甘油=900 μL:100 μL的比例混合, -70 ℃液氮中保存过夜。复苏细胞时, 在37 ℃水浴中迅速解冻细胞后加入适量的完全培养液。5 h-8 h后更换完全培养液并常规传代培养。

### 1.7 CTC模型的建立及细胞捕获效率实验

制备NSCLC细胞系A549细胞、HCC827细胞、NCI-H1650细胞株、NCI-H3122细胞株的单细胞悬液, 经计数后分别以50、100、200、500、1, 000共5个细胞数量梯度加入7.5 mL正常人全血中, 模拟CTC, 检测制备的磁球捕获CTC的能力。将待测模型的各分组细胞样品进行1, 000 rpm/min离心10 min; 小心取中上层液置于EP管中, 加入与其等量的PBS充分混匀。随后加入EGFR和Vimentin及FA纳米脂质磁球20 μL, 室温下孵育15 min, 每5 min混匀1次; 将离心管插入磁分离架吸附10 min, 吸弃上清液后加入10 μL 4%的多聚甲醛固定细胞10 min; 使用PBS溶液对固定处理的捕获CTC进行磁分离洗涤2次; 加入10 μL FITC标记的CK19单克隆抗体(CK19-FITC)、20 μL DAPI染色液、10 μL PE标记的CD45抗体(CD45-PE), 混匀后避光染色15 min; 染色结束后, 磁分离5 min, 用去离子水洗涤两次, 充分洗掉未结合的抗体和DAPI; 最后向离心管中加入15 μL去离子水重选CTC, 混匀后的液体均匀涂于多聚赖氨酸处理的防脱载玻片, 待液滴干于荧光显微镜下观察计数。

### 1.8 纳米脂质磁球对肺癌临床血样中CTC的分离鉴定

收集NSCLC患者7.5 mL抗凝血液, 1, 000 rpm离心10 min; 小心取中上层液置于EP管中, 加入与其等量的PBS充分混匀; 加入免疫脂质磁球20 μL, 室温孵育30 min, 每10 min混匀一次; 将EP管插入磁分离架上吸附5 min, 吸弃上清液, 加入10 μL 4%的多聚甲醛固定细胞10 min; PBS洗涤3次; 加DAPI染色液30 μL、CK19-FITC染色液10 μL、CD45-PE染色液10 μL混匀避光染色15 min; PBS洗涤3次; 向EP管中加入15 μL去离子水重悬, 均匀涂于防脱载玻片, 待液滴干后荧光显微镜下观察计数。

### 1.9 统计学分析

采用SPSS 18.0统计学软件进行统计分析, 正态分布计量资料以均数±标准差(Mean±SD)表示, 重复测量数据比较采用重复测量设计的方差分析, 组间比较采用单因素方差分析, *P* < 0.05表示差异有统计学意义。

## 2 结果

### 2.1 免疫磁球的制备与肺癌CTC检测

免疫磁性微球的制备流程见图 1。纳米磁球系统由5种功能元素组成：(ⅰ)EGFR抗体衍生物、(ⅱ)氧化铁(Fe_3_O_4_)纳米颗粒、(ⅲ)Cholesterol及(ⅳ)DOPC。EGFR抗体用作生物配体, 以特异性捕获在其膜上过表达EGFR受体的癌细胞亚群。超顺磁性Fe_3_O_4_纳米颗粒允许EGFR抗体捕获细胞后的磁性分离, 并且分离的CTC可以用免疫荧光进行鉴定。Vimentin和FA磁球的制备与EGFR相同。制备的蛋白抗体免疫磁性微球加入到肺癌患者外周血中, 捕获的肺癌CTC通过免疫荧光进行鉴定后计数。

**1:**
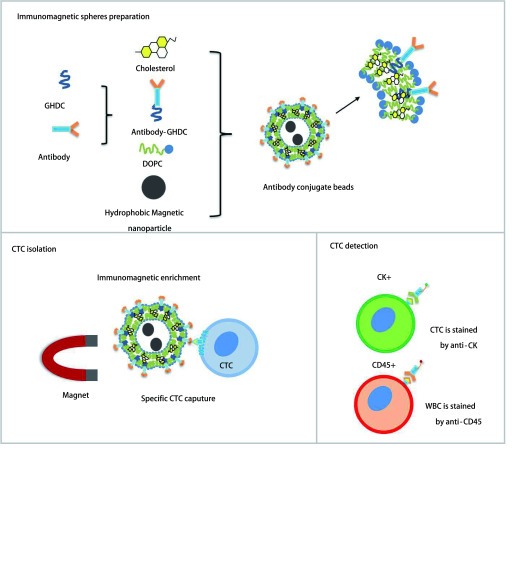
免疫磁球的制备及CTC分离鉴定流程图 Immunomagnetic spheres preparation and CTC separation and identification flowchart. WBC: white blood cell.

### 2.2 免疫磁球的材料学表征及性能评价

Vimentin磁球的平均粒径为491 nm, Zeta电位为+20.4 mv。FA磁球的平均粒径为359 nm, Zeta电位为+16.5 mv。EGFR磁球的平均粒径为436 nm, Zeta电位为+29.7 mv。本研究制备的磁球具有较小的粒径和较高的稳定性。EGFR、Vimentin和FA磁球的原子力显微镜观察结果见图 2A, 由图可知, 三种免疫磁性微球为大小不一的球形, 没有发生团聚现象, 说明免疫磁性微球稳定性较好, 形状较规则, 大小在200 nm左右, 具有脂质体的囊泡特性。从图 2B中的紫外测试图中可以发现, 三种抗体免疫磁球约在276 nm处出现一个宽的吸收峰, 表明磁球表面上确实接上了EGFR、Vimentin和FA。从图 2C中的普鲁士蓝染色结果可以明显看到细胞表面呈现蓝色。这是由于细胞表面包覆的免疫磁球被铁质的特异性染料-普鲁士蓝染色。由图 2C可知, 本研究制备的三种免疫磁球与肺癌A549细胞具有较好的亲和性。三种磁球对不同肺癌细胞系A549细胞、HCC827细胞、NCI-H1650细胞株、NCI-H3122细胞株的捕获效率如图 3所示。磁球对不同肺癌细胞系具有较为稳定的回收效率, EGFR、Vimentin和FA磁球平均捕获效率分别为78.0%、79.0%和82.0%, 同时使用三种磁球捕获效率为91.0%。以上结果表明EGFR、Vimentin和FA磁球具有较高的肺癌细胞亲和能力和比较稳定的捕获能力。

**2:**
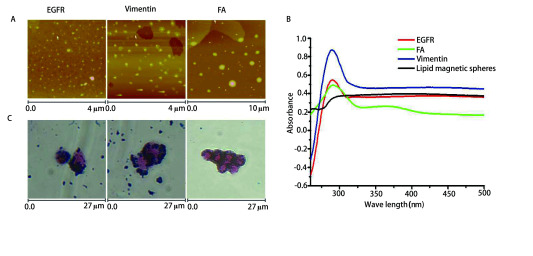
Vimentin、FA、EGFR免疫脂质磁球的表征。A：三种磁球的原子力显微镜观察图片; B：三种磁球的紫外吸收光谱图; C：三种磁球的普鲁士蓝染色图片。 Characterization of Vimentin, FA, EGFR immunolipid magnetic spheres. A: Atomic force microscope observation picture of three magnetic spheres; B: Ultraviolet absorption spectra of three magnetic spheres; C: Prussian blue stained picture of three magnetic spheres; FA: folic acid; EGFR: epidermal growth factor receptor.

**3:**
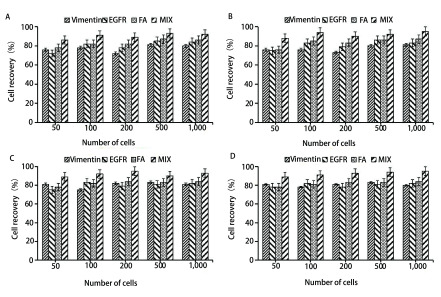
三种磁球(Vimentin、FA、EGFR)及其混合物对不同肺癌细胞的捕获效率。A：A549细胞; B：HCC827细胞; C：NCI-H1650细胞; D：NCI-H3122细胞。 Capture efficiency of three kinds of magnetic spheres (Vimentin, FA, EGFR) and their mixtures on different lung cancer cells. A: A549 cells; B: HCC827 cells; C: NCI-H1650 cells; D: NCI-H3122 cells.

### 2.3 肺癌临床血样中循环肿瘤细胞的免疫荧光鉴定

将EGFR、Vimentin和FA免疫磁球分离出来的肺癌患者的CTC进行涂片观察, 其中在白光下具有明显细胞形态, CK19-FITC绿色荧光为强阳性, DAPI蓝色荧光为强阳性, 2种荧光叠加后重合, 且CD45染色不显示荧光的细胞判定为肺癌CTC, 如图 4所示。

**4:**
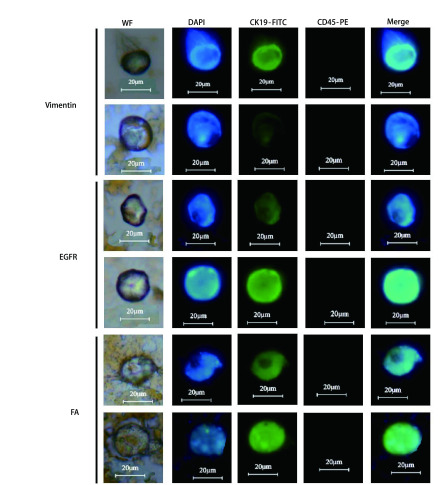
Vimentin、EGFR、FA三种免疫脂质磁球捕获临床血样中CTC的荧光显微镜观察结果。 Vimentin, EGFR, and FA immunolipid magnetic spheres captured CTC in clinical blood samples for fluorescence microscopy observations.

### 2.4 CTC数量与患者的临床特征的关系

在60例NSCLC患者中, 每个肺癌患者血液中均能检测到CTC, 记CTC≥2为阳性, 则CTC检测阳性率为100.0%。图 5A是单独分别使用三种磁球及联合使用三种磁球对60例患者的CTC数量进行检测, 结果显示使用单一磁球捕获肺癌患者外周血中CTC时, 三种磁球中叶酸磁球捕获效果最好, 而联合使用三种磁球捕获CTC效果更加显著。使用EGFR、Vimentin和FA单一磁球分别捕获和联合使用三种磁球捕获肺癌患者外周血中CTC, 若以CTC≥2为CTC阳性, 得到的CTC阳性率结果如图 5B所示, EGFR、Vimentin和FA捕获的阳性率分别为65.0%、33.3%和93.3%, 联合使用三种磁球捕获的阳性率为100.0%。与临床信息的相关性分析见表 1, 联合使用三种磁球检出的CTC数量与临床分期具有相关性。

**5:**
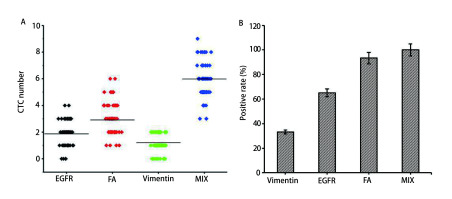
三种磁球及其混合物捕获的CTC数量与阳性率。A：三种磁球及其混合物捕获肺癌患者血样中的CTC数量统计; B：三种磁球及其混合物对肺癌患者CTC进行捕获, 以为CTC≥2为阳性, 统计CTC阳性率。 The number and positive rate of CTC captured by the three magnetic spheres and their mixtures. A: Statistics of CTC in blood samples of lung cancer patients captured by three magnetic spheres and their mixtures; B: Three magnetic spheres and their mixtures were used to capture CTC from patients with lung cancer. CTC≥2 was positive, and CTC positive rates were counted.

**1:** 磁球混合物检出CTC总数与肺癌患者临床病理特征的相关性 Correlation between total CTC and clinicopathological characteristics of lung cancer patients detected by magnetic ball mixture

Characteristic	Number of cases (%)	CTC total		*P*
		< 5	≥5	
Gender				
Male	39	4	35	> 0.05
Female	21	2	19	
Age (yr)				
< 50	24	2	22	> 0.05
≥50	36	4	32	
Tumor size (cm)				
< 3	15	2	13	> 0.05
3-7	34	3	31	
> 7	11	1	10	
Clinical staging (TNM)				
Ⅰ	9	5	4	< 0.05
Ⅱ	12	1	11	
Ⅲ	25	0	25	
Ⅳ	14	0	14	
TNM: tomor-node-metastasis; CTC: circulating tumor cell.				

## 3 讨论

CTC检测为癌症患者的辅助诊断, 治疗效果评估和评价预后提供了一种新方法^[22]^。EpCAM已被广泛的用于上皮型CTC的分离富集, 然而CTC从组织进入血液传播会发生上皮-间质转化(epithelial-mesenchymal transition, EMT), 从而导致肿瘤发生转移、耐药和免疫逃避等, 以EpCAM作为主要的捕获工具的富集方式将影响EMT细胞的捕获, 导致假阴性率提高。肿瘤细胞的上皮型标示物(CK, EpCAM, E-cad)与间质型标示物(Vimentin, N-cad, O-cad)经历着此消彼涨的动态变化, 以EMT阶段为例, CTC的间质型蛋白呈现强表达, 而上皮型蛋白则为弱或无表达^[23]^。通过选用Vimentin作为间质型CTC标志物, 采用Vimentin磁球捕获高表达Vimentin的CTC, 我们发现Vimentin磁球对肺癌细胞株各细胞株的平均捕获效率达到79%。

肿瘤细胞中存在EGFR信号传导通路, 如EGFR过度表达、*EGFR*突变等的异常, 从而促进细胞不断增殖并抑制其凋亡, 导致生长调节失控。*EGFR*突变型患者对EGFR-酪氨酸激酶抑制剂(tyrosine kinase inhibitor, TKIs)类药物的有效率高, 副作用轻, 耐受性好, 已成为晚期NSCLC的一线治疗药物。目前利用外周血循环肿瘤细胞检测EGFR表达及突变已成为肺癌领域研究的热点^[24, 25]^。我们选用EGFR磁球捕获高表达EGFR的CTC, 发现EGFR磁球对肺癌各细胞株的平均捕获效率达到78%。

FA是一种细胞表面糖蛋白, 在多种癌症中高度表达, 尤其是在卵巢癌和肺癌中, 其广泛的被应用于CTC的分离富集^[26, 27]^。我们选用FA磁球捕获高表达FA的CTC, 发现FA磁球对肺癌各细胞株的捕获效率达到82%。通过EGFR、Vimentin、FA三种免疫脂质体磁球特异性捕获EGFR、Vimentin、FA三种分型的CTC, 它们对肺癌各细胞株的平均捕获效率分别为78.0%、79.0%、82.0%, 结果表明单独使用三种免疫脂质磁球时, FA磁球有一个相对较高的捕获效率, 联合使用三种磁球能够达到一个更高的捕获效率, 平均捕获效率为91.0%。将三种免疫脂质体磁球应用于60例NSCLC患者中, 对于我们建立的这套CTC分选系统, 我们使用每7.5 mL血液中有2个CTC为cutoff值, EGFR、Vimentin、FA阳性率分别为65.0%、33.3%、93.3%, 而联合使用三种磁球捕获CTC的阳性率为100.0%, 结果表明联合使用三种磁球对CTC有更高的检出率, 最后, 我们将联合使用三种磁球检出的CTC总数与患者临床信息进行相关性分析, 采用5为分界值, 发现联合使用三种磁球检出的CTC数量与临床分期具有相关性。

我们利用基于EGFR、Vimentin、FA免疫脂质体的磁性纳米系统能够有效捕获EGFR+、Vimentin+、FA+过表达CTC, 同时能够从外周血细胞中分离捕获的细胞进行免疫荧光鉴定, 验证我们的实验结果。

本研究证实通过联合使用EGFR、Vimentin和FA三种免疫脂质磁球作为CTC捕获的靶点, 用以增加NSCLC CTC检测的敏感性是新型实用的检测手段。通过这个策略, 更多异质性CTC能够被捕获, 可用于早期癌症的诊断及其复发评估, 同时发现检出的CTC数量与临床分期具有相关性。也正因为捕获过程中并不会破坏循环肿瘤细胞的细胞膜, 所以利用该方法捕获的CTC能够用于进一步的分析, 包括单细胞测序等。尽管本实验得到了一个很有希望的结果, 但是其临床有效性仍需进一步验证。
